# Hematological cancer patients’ social support, coping strategies, anxiety, depression and posttraumatic growth: a structural equation model

**DOI:** 10.3389/fonc.2025.1540973

**Published:** 2025-05-14

**Authors:** Taoyun Liang, Ling Mao, Xinwen Du, Fengjiao Chen

**Affiliations:** West China Hospital, Sichuan University, Chengdu, China

**Keywords:** hematologic neoplasms, posttraumatic growth, coping skills, social support, anxiety, depression

## Abstract

**Objective:**

Posttraumatic growth (PTG), defined as positive psychological changes following traumatic events, has been observed in some hematological cancer patients during their disease course. These changes, encompassing shifts in life perspective, interpersonal relationships, and self-perception, are critical for psychological recovery. However, the interplay of social support, coping strategies, anxiety, and depression in shaping PTG remains unclear. Therefore, the aim of this study was to explore these associations in hematological cancer patients using a hypothetical model.

**Methods:**

From August 2019 to May 2021, a cross-sectional survey was conducted with 474 hospitalized patients with hematological cancer at West China Hospital, Sichuan University, China (a tertiary hospital). The Social Support Rating Scale, Medical Coping Modes Questionnaire, Hospital Anxiety and Depression Scale, and Posttraumatic Growth Inventory were used for data collection. Correlation and regression analyses were performed using SPSS 26.0, a structural equation model was constructed using AMOS 24.0 software, and the confidence interval of the mediating effect was calculated using the bias-corrected bootstrap method.

**Results:**

Social support was positively associated with PTG in hematological cancer patients (*β* = 0.564, *P* = 0.004). Avoidance (*β* = 0.199, *P* = 0.034) and acceptance–resignation (*β* = -0.315, *P* = 0.002) coping strategies mediated this association, with depression (*β* = -0.123, *P* = 0.009) further mediating the effects of coping strategies on PTG.

**Conclusion:**

These findings provide a basis for further research on PTG in cancer patients, particularly with respect to coping strategies in various dimensions. Enhancing social support and addressing maladaptive coping may promote PTG. Tailored interventions targeting depression management and culturally sensitive support systems are recommended to enhance PTG.

## 1 Introduction

Hematological cancers, including leukemia, lymphoma, and multiple myeloma, constitute one of China’s top ten cancer categories in terms of incidence and mortality (1). Although therapeutic advancements have increased survival rates (2), a diagnosis of hematological cancer is often associated with significant physical and psychological burdens. While cancer diagnoses are universally recognized as profoundly distressing life events for many patients (3), emerging evidence indicates that some patients develop posttraumatic growth (PTG), a transformative process characterized by positive psychological shifts in life perspectives, interpersonal dynamics, and self-concept following adversity (4). The growing emphasis on holistic health paradigms underscores the imperative to elucidate PTG mechanisms in oncological populations.

PTG represents a beneficial psychological outcome arising from engagement with traumatic events and is operationalized as positive cognitive-emotional transformations stemming from efforts to address existential challenges (5). These changes manifest across three domains: life perspective, interpersonal relationships, and self-perception (6). As a critical construct in survivorship research (7), PTG development involves complex interactions between intrapersonal factors (e.g., cognitive appraisal, coping mechanisms) and environmental resources (8). While social support and adaptive coping have been identified as PTG facilitators in solid tumor research (e.g., breast cancer) (9, 10), hematological cancer patients face distinct challenges, including protracted treatment regimens (e.g., hematopoietic stem cell transplantation) and greater relapse risks (11), potentially engendering differential psychological trajectories. Notably, PTG mechanisms in this population remain underexplored, particularly with respect to the interplay among social support, multidimensional coping strategies, affective states (anxiety/depression), and PTG development.

Social support, conceptualized as the provision of emotional, informational, and tangible assistance through interpersonal networks, encompasses received support, perceived availability, and satisfaction with support adequacy (12). Perceived support has particularly robust associations with mental health outcomes—a focal point of this investigation. Empirical evidence positions social support as a protective factor against trauma-related psychopathology (13), with oncology studies consistently linking support systems to PTG emergence (14–16). For example, stable support networks mitigate isolation in breast cancer patients (14), facilitate cognitive-emotional processing in rectal cancer patients (15), and promote adaptive coping during stem cell transplantation (16).

Coping strategies, defined as cognitive-behavioral efforts to manage internal and external stressors (17), have multidimensional influences on PTG (18, 19). While active strategies such as treatment engagement and optimism cultivation may foster resilience (20), medical coping modes transcend simplistic positive/negative dichotomies (21). Feifel’s tripartite model delineates disease-specific strategies: confrontation (active problem-solving), avoidance (actively avoiding the emergency situation of the disease), and acceptance–resignation (passive resignation from the disease) (22). Theoretical frameworks posit differential PTG associations: confrontation may promote growth through challenge mastery (10); avoidance might enable psychological adjustment through temporary distress reduction (23); and acceptance–resignation potentially impedes growth via perceived helplessness (24). Empirical findings remain contradictory, with studies reporting positive avoidance-PTG associations in gynecological cancers (10), and negative correlations in colorectal cancer populations (25), highlighting the necessity for population-specific investigations.

The affective landscape of hematological cancer patients frequently includes anxiety and depression (26). While some studies have identified inverse relationships between depression/anxiety and PTG (27, 28), others have identified positive anxiety-PTG associations in breast cancer cohorts (29). This divergence suggests bidirectional emotion–growth dynamics, where psychological distress may paradoxically catalyze meaning-making processes. Emerging evidence indicates that targeted psychotherapeutic interventions can enhance PTG despite persistent affective symptoms (30), underscoring the need to clarify these complex interrelationships.

Current gaps in PTG research on hematological cancer are threefold: (1) insufficient examination of the of role social support, (2) limited understanding of multidimensional coping strategy effects, and (3) unclear anxiety/depression–PTG dynamics. Clinical distinctiveness—including prolonged hospitalization, treatment-related complications (e.g., immunosuppression, hemorrhagic risks), and intensive therapies (11) — necessitates specialized investigation. Therefore, the aim of this study was to explore the effects of social support on the PTG of patients with hematological cancers; analyze the mediating effects of various coping strategies, anxiety, and depression; and provide a basis for more effective support and interventions to help patients actively cope, adapt, and grow.

## 2 Materials and methods

### 2.1 Hypothesized model

**Figure 1:**
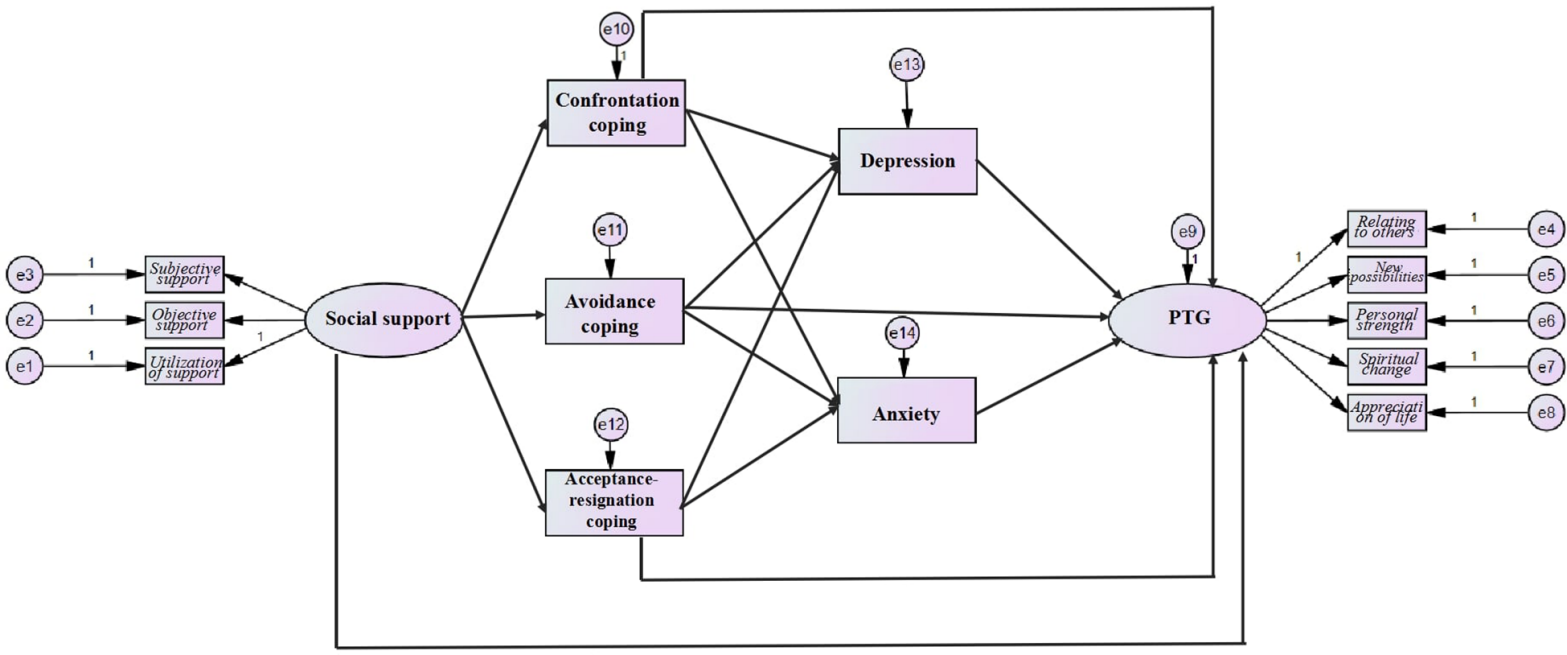
Hypothesized model of the interrelationships among social support, coping strategies, anxiety, depression and PTG.

Structural equation modeling (SEM) represents an advanced statistical approach for evaluating complex interrelationships among observed and latent variables, enabling simultaneous examination of direct and indirect pathways within theoretical frameworks (31). This methodology is particularly suited to our investigation, as it accommodates the multidimensional nature of psychosocial constructs while accounting for measurement error. By operationalizing social support, coping strategies (confrontation, avoidance, acceptance–resignation), affective states (anxiety/depression), and PTG as distinct yet interconnected dimensions, SEM allows for a nuanced analysis of their systemic interactions. Guided by prior empirical and theoretical work, we formulated five hypotheses to test the proposed multiple-mediation model (**Figure 1**):

#### 2.1.1 H1

Social support is directly positively associated with PTG.

#### 2.1.2 H2

Social support is associated with coping strategies, with variations across strategy types.

#### 2.1.3 H3

The presence of three coping strategies is associated with depression/anxiety and PTG.

#### 2.1.4 H4

Depression and anxiety are associated with PTG.

#### 2.1.5 H5

Coping strategies and emotional states mediate the social support–PTG association.

### 2.2 Study design and participants

We conducted a cross-sectional study involving hematological cancer patients hospitalized at West China Hospital, Sichuan University, China, from August 2019 to May 2021. We approached a total of 743 patients, and 629 eligible patients were invited to participate in the study. Among the patients invited, 505 completed the questionnaire (80.29% response rate). We excluded 31 questionnaires that were incomplete, exhibited uniform responses across all the items, or contained logical inconsistencies. Only complete data were received, and 474 valid questionnaires were ultimately returned, for a valid return rate of 93.86%. The criteria for selecting the participants were as follows: 1) were diagnosed with leukemia, lymphoma or multiple myeloma; 2) were age ≥ 18 years; 3) had adequate Chinese reading and writing ability to finish the survey; and 4) were willing to participate. The exclusion criteria were as follows: 1) concurrent treatment for other cancers; 2) in the terminal phase of the disease; and 3) mental or physical inability to participate in the study. The terminal phase of the disease refers to the stage where patients are receiving end-of-life care or palliative treatment, with no further curative treatment options available. Most of these patients were excluded because their physical condition did not allow them to complete the questionnaire. Owing to the settings of the survey, for patients who were hospitalized more than once within the study duration, their participation in the survey was be limited to a single instance.

### 2.3 Data collection

In the present study, we implemented a standardized data collection protocol using anonymized paper questionnaires to ensure methodological consistency. Two research assistants underwent structured training: (1) comprehensive review of ethical guidelines and study objectives; (2) standardized patient interaction protocols; and (3) competency assessments in neutral survey administration. This training emphasized maintaining participant autonomy while addressing queries about item interpretation without introducing response bias.

Following eligibility confirmation through medical record screening, enrolled hematology inpatients received structured briefings detailing the study purposes, potential risks/benefits, and data protection measures. Written informed consent was obtained prior to questionnaire distribution, emphasizing the participants’ right to withdraw without consequence. The participants were then asked to complete the printed questionnaires independently in a private setting to ensure confidentiality and minimize external influences. The questionnaires were designed to take approximately 15–20 minutes to complete. Research assistants were available to provide assistance if participants had any difficulties understanding the questions or required clarification.

To ensure data quality, completed questionnaires were reviewed immediately for completeness and accuracy. Any missing or ambiguous responses were addressed with the participants while they were still present. The data were entered into a secure database, with double-checking to minimize entry errors. All the data were anonymized and stored securely in accordance with the ethical guidelines of the study.

### 2.4 Ethical considerations

Ethical oversight was secured through formal approval by the Biomedical Ethics Review Committee, West China Hospital, Sichuan University (HXHL19039), with strict adherence to the Declaration of Helsinki. Prior to enrollment, eligible participants received comprehensive verbal and written briefings detailing the study objectives, voluntary participation rights, and potential psychological risks. Written informed consent was obtained to ensure autonomous decision-making.

As part of the ethical safeguards, all participants underwent systematic psychological screening using the Hospital Anxiety and Depression Scale (HADS). Individuals scoring ≥11 on either subscale—indicating clinically significant symptoms—were provided priority referrals to licensed clinical psychologists affiliated with the hospital’s psychosocial oncology unit. For participants exhibiting acute distress during PTG assessments (e.g., tearfulness, verbalized helplessness), immediate onsite counseling was administered by trained mental health professionals.

These support mechanisms were explicitly outlined in the consent documentation, emphasizing 24/7 availability throughout the study period. All data anonymization procedures adhered to strict confidentiality protocols, with encrypted identifiers ensuring the irreversible delinking of personal health information from research data.

### 2.5 Measures

#### 2.5.1 Informational questionnaire for demographic characteristics

After referring to previous literature and expert consultation, we identified the following demographic information as the study variables: age, sex, marital status, educational status, type of work, diagnosis, transplantation, recurrent or refractory diseases and previous stressful events. Stressful events refer to significant life changes or experiences that have caused considerable emotional, psychological, or physical strain. This may include but is not limited to major life changes (e.g., divorce, job loss), traumatic experiences (e.g., accidents, violence), or chronic stressors (e.g., long-term illness, financial difficulties) (32). We assessed exposure to stressful events using a single-item yes/no question.

#### 2.5.2 PTG

PTG was assessed using the Chinese version of the Posttraumatic Growth Inventory (PTGI-C) (33). This 21-item scale comprises five subscales: relating to others, new possibilities, personal strength, spiritual change, and appreciation of life. Each item is scored on a 6-point Likert-type scale ranging from 0 (“no change”) to 5 (“extreme change”). There are no reverse-scored items, and the total score is obtained by summing the scores of all the items. Scores range from 0 to 105, with higher scores indicating greater PTG. The PTGI-C was developed through a rigorous translation and cultural adaptation process based on the original scale by Tedeschi and Calhoun (34). The translation involved a forward-backward translation procedure to ensure semantic equivalence between the original and Chinese versions. The cultural adaptation process included pilot testing with a sample of Chinese trauma survivors to ensure that the items were culturally appropriate and comprehensible. The content validity index (CVI) was calculated on the basis of expert reviews, resulting in a CVI of 0.980, indicating excellent content validity. The internal consistency reliability of the PTGI-C was assessed using Cronbach’s α, which was found to be 0.920, suggesting high reliability. These validation steps ensure that the PTGI-C is a reliable and valid instrument for measuring PTG in the Chinese population.

#### 2.5.3 Social support

Social support was assessed using the Social Support Rating Scale (SSRS) (35). This 10-item scale comprises three dimensions: subjective support (items 1-4, and 8-10; scored on a 4-point Likert scale from 1 [lowest] to 4 [highest]), objective support (items 6-7; scored based on a 9-source checklist), and utilization of support (item 5; five subitems scored on a 4-point scale). The total score ranges from 12 to 66 points, with higher scores indicating stronger social support. The SSRS was developed through extensive research within the Chinese population to capture the unique aspects of social support in this cultural context. It has undergone rigorous validation, including pilot testing and cultural adaptation, to ensure its applicability in Chinese settings (36). The internal consistency reliability of the SSRS is robust, with Cronbach’s α values ranging from 0.83 to 0.90 for the individual subscales and the overall scale. These validation steps confirm that the SSRS is a reliable and valid instrument for measuring social support in the Chinese context.

#### 2.5.4 Coping strategies

Coping strategies were assessed using the Chinese version of the Medical Coping Modes Questionnaire (MCMQ) (37), a 20-item scale adapted from the original scale by Feifel et al. (22). The MCMQ measures three dimensions: confrontation (active problem-solving), avoidance (cognitive-behavioral disengagement), and acceptance–resignation (passive acquiescence). Each item is scored on a 4-point Likert scale ranging from 1 (“never”) to 4 (“always”), with eight reverse-scored items (items 1, 4, 9, 10, 12, 13, 18, and 19). Higher scores in each dimension indicate a greater inclination toward a particular coping strategy. The Chinese version of the MCMQ was developed through a rigorous translation and cultural adaptation process. The translation involved a forward-backward translation procedure to ensure semantic equivalence between the original and Chinese versions. The cultural adaptation included pilot testing with a sample of 50 Chinese patients to ensure that the items were culturally appropriate and comprehensible. The CVI was calculated on the basis of expert reviews and was 0.90, indicating good content validity. The internal consistency reliability of the MCMQ was assessed using Cronbach’s α, with values of 0.690, 0.600, and 0.760 for the confrontation, avoidance, and acceptance–resignation dimensions, respectively. These validation steps ensure that the MCMQ is a reliable and valid instrument for measuring coping strategies in the Chinese population.

#### 2.5.5 Anxiety and depression

The Hospital Anxiety and Depression Scale (HADS) (38) was used to assess the patients’ anxiety and depression levels. The HADS is a self-evaluation scale that includes two subscales, anxiety (HADS-A) and depression (HADS-D), with a total of 14 items. Seven items rate depression, and seven items rate anxiety. Each item has four options, ranging from never to always, scored from 0 to 3 points. There are six reverse-scored items, five on the depression subscale and one on the anxiety subscale. The total score range for anxiety and depression is 0–21 points. Scores of 0–7 are classified as asymptomatic, 8–10 as suspicious symptoms, and 11–21 as definitely symptomatic. The HADS was translated and culturally adapted for use in the Chinese population. The CVI was calculated on the basis of expert reviews and was 0.91, indicating good content validity. The internal consistency reliability of the HADS was assessed using Cronbach’s α, with values of 0.879 for the overall scale, 0.806 for the anxiety subscale, and 0.806 for the depression subscale (39). These validation steps ensured that the HADS is a reliable and valid instrument for measuring anxiety and depression in the Chinese population.

### 2.6 Data analysis

G*Power is a versatile tool designed for computing statistical power and determining the necessary sample size for researchers conducting specific statistical tests. Given the inherent complexity of conducting power analyses for SEM, we opted for a simplified approach based on multiple regression techniques. The overall sample size for this study was determined to ensure adequate statistical power for detecting medium effect sizes, as defined by Cohen (40) (*f²*= 0.15) in multiple regression analyses. Our study includes 7 predictors. Uing G*Power version 3.1, we calculated that a total of 153 participants would be required to achieve a power level of 95%, with an α criterion of 0.05. To account for an anticipated dropout rate of 25%, we established a minimum sample size of 192 participants for the study. Given that the SEM encompasses more intricate models and a greater number of parameter estimates, additional considerations are essential. Reflecting on the model’s complexity and the potential for measurement errors, as suggested by reference (41), a minimum of 300 samples is advisable when seven or fewer parameters are estimated within an SEM framework. This ensures that the analysis is robust and that the results are reliable. Our sample size assumption was based on a combination of statistical power analysis, consideration of the model’s complexity, the number of predictors, an anticipated dropout rate, and recommendations from the methodological literature. These factors collectively informed our decision to calculate a sample size that would provide a solid foundation for a reliable and robust analysis.

All analyses were carried out using SPSS (version 26.0, SPSS, Inc., Chicago) and AMOS (version 24.0, SPSS, Inc., Chicago). First, the data were preliminarily analyzed with SPSS. The data for social support, coping strategies, anxiety, depression and PTG followed a normal distribution. The correlations among the five variables of social support, coping strategies, anxiety, depression and PTG were explored using Pearson correlation analysis. SEM was subsequently conducted with AMOS 24.0 to test the hypothesis model. The parameters used for evaluating the fitting effect of the models were as follows: 1) CMIN/df ≤ 3.00, 2) root mean square error approximation ≤ 0.08, 3) comparative fitting index ≥ 0.90, 4) goodness-of-fit index ≥ 0.90, and 5) higher parsimonious normed fit index (31). Additionally, the bootstrap method test with percentile bias correction was used to examine the mediating effects of three coping strategies (confrontation, avoidance, and acceptance–resignation), anxiety and depression. Furthermore, to examine the effect of social support on PTG, the 95% confidence intervals for bias-corrected percentile bootstrapping through a bootstrapped sample of 5000 were calculated (42). A two-tailed *P* value <0.05 was considered to indicate statistical significance.

## 3 Results

### 3.1 Participant characteristics

**Figure 2:**
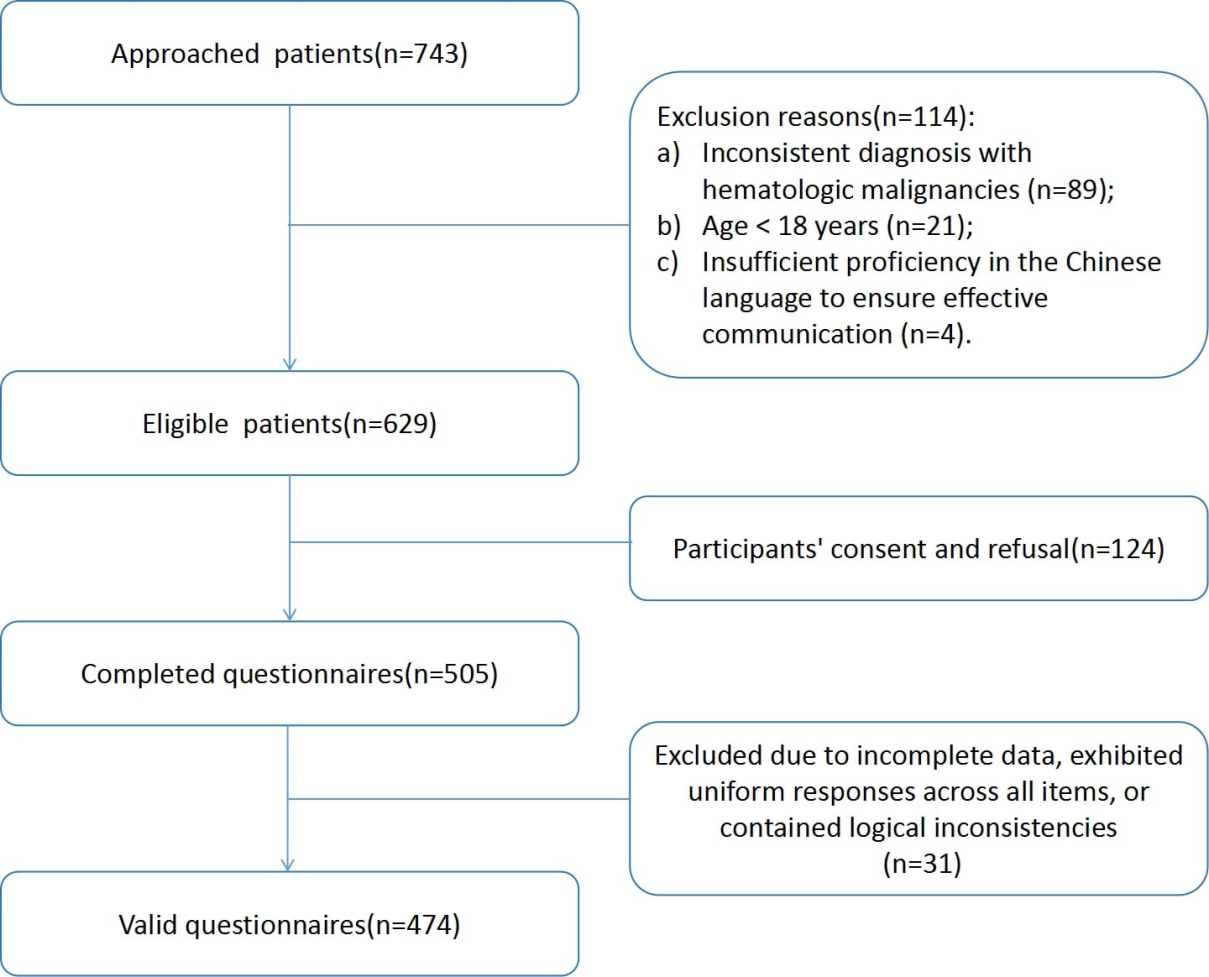
Flow chart of patients.

A total of 474 valid questionnaires were included in the analysis(valid return rate of 93.86%) (**Figure 2**). **Table 1** shows the ages of the 474 patients with hematological cancer who participated in the study; the majority were aged 41–60 years old (42.2%), were married (77.0%), and were male(53.2%). The educational status of the patients was as follows: 15.4% had completed primary school, 26.4% had completed middle school, 18.1% had a high school or technical secondary school diploma, 17.5% had a junior college degree, and 22.6% had a bachelor’s degree or higher. Acute leukemia was the most common form of hematological cancer (62.3%), followed by lymphoma (29.1%), multiple myeloma (5.3%), and chronic leukemia (2.3%). Among the patients, 17.3% had recurrent or refractory disease. Bone marrow transplantation was not performed in 92.4% of the patients. A total of 21.3% of the patients experienced a stressful event. **Table 1** shows the characteristics of the 474 participants.

**Table 1:** Demographic and clinical characteristics of the participants (N = 474).

Constant	N	Percentage (%)
Age (Years)		
18~40	189	39.9
41~60	200	42.2
>60	85	17.9
Sex		
Male	252	53.2
Female	222	46.8
Marital status		
Unmarried	99	20.9
Married	365	77.0
Divorce/Widowed	10	2.1
Educational status		
Primary school	73	15.4
Middle school	125	26.4
High school/technical secondary school	86	18.1
Junior college	83	17.5
Bachelor degree or above	107	22.6
Diagnosis		
Multiple myeloma	25	5.3
Acute leukemia	300	62.3
Lymphoma	138	29.1
Chronic leukemia	11	2.3
Recurrent or refractory diseases		
Yes	82	17.3
No	392	82.7
Accepting transplantation		
Yes	36	7.6
No	438	92.4
Previous stressful events		
Yes	102	21.5
No	372	78.5

### 3.2 Descriptive statistics and correlations of social support, PTG, coping strategies, anxiety, and depression

**Table 2** presents the descriptive statistics and correlations of the key variables: social support, PTG, coping strategies (confrontation, avoidance, and acceptance–resignation), anxiety, and depression. PTG was positively correlated with social support, confrontation, and avoidance coping strategies and negatively correlated with acceptance–resignation coping, anxiety, and depression.

**Table 2:** Descriptive statistics and correlations of the key variables (N = 474).

Variable	Mean ± Standard deviation	1	2	3	4	5	6	7
1 PTG	64.96 ± 19.585							
2 Social support	43.12 ± 7.650	0.158**						
3 Confrontation coping	18.89 ± 3.577	0.234**	0.255**					
4 Avoidance coping	16.64 ± 2.525	0.364**	0.134**	0.322**				
5 Acceptance–resignation coping	8.74 ± 2.839	-0.243**	-0.135**	-0.025	0.042			
6 Anxiety	8.93 ± 2.492	-0.104*	-0.078*	0.064	0.47	0.490**		
7 Depression	13.00 ± 2.943	-0.294**	-0.204**	-0.143**	-0.143**	0.527**	0.517**	

*P < 0.05. **P < 0.01.

### 3.3 Hypothesized model

The initial structural equation model demonstrated suboptimal fit indices (**Table 3**), with nonsignificant associations identified between confrontation coping strategies and anxiety (*β* = 0.053, *P* = 0.071), avoidance coping strategies and anxiety (*β* = 0.001, *P* = 0.974), and anxiety and PTG (*β* = 0.094, *P* = 0.365).

**Table 3:** Hypothetical model and adjusted model parameters.

	CMIN			RMSEA 95%CI							
Model	*χ^2^*	df	*χ* ^2^/df	RMSEA	Lower bound	Upper bound	GFI	AGFI	CFI	TLI	IFI
Acceptable threshold	*-*	*-*	*≤3.00*	*≤0.08*	*-*	*-*	*≥0.90*	*≥0.90*	*≥0.90*	*≥0.90*	*≥0.90*
Hypothetical model	297.494	55	5.409	0.097	0.079	0.100	0.911	0.853	0.901	0.860	0.860
Adjusted model	163.339	52	2.545	0.067	0.056	0.079	0.951	0.914	0.955	0.932	0.932

CMIN, Minimum discrepancy; CI, Confidence interval; df, Degree of freedom; χ^2^, Chi square fitting statistics; RMSEA, Approximate root mean square error; GFI, Goodness of fit index; AGFI, Adjusted goodness of fit index; CFI, Comparative Fit Index; TLI, Tucker-Lewis index; IFI, Incremental fit index.

The modification index (MI) and Parameter change (Par Change) are used to evaluate model fit and suggest potential modifications. Further analysis revealed substantive modification indices (MI > 4.0), suggesting covariances between latent constructs. Those indices were as follow:

confrontation and acceptance–resignation coping strategies (MI = 36.519; Par Change = 2.367), avoidance and confrontation strategies (MI = 5.041, Par Change = 0.711), and anxiety and depression strategies (MI = 65.785, Par Change = 1.966). Measurement model refinements were indicated for covariances between the following: subjective and objective social support components (MI = 8.918, Par Change = 2.077); specific PTG dimensions: relating to others with personal strength (MI = 6.970, Par Change = 1.169); relating to others with spiritual change (MI = 8.785, Par Change = -0.899); new possibilities with spiritual change (MI = 5.787, Par Change = 0.453); and appreciation of life with spiritual change (MI = 4.769, Par Change = 0.537).

On the basis of these findings, the hypothetical model was revised to improve model fit. The revised model demonstrated acceptable fit indices, confirming the appropriateness of these modifications.

### 3.4 Adjustment model

After model pruning and restandardization, the adjusted model shown in **Figure 2** and **Table 3** has acceptable model fitting parameters. Social support was positively related to confrontation and avoidance and negatively related to acceptance–resignation. Confrontation and avoidance coping strategies were negatively correlated with depression, and avoidance strategies were negatively correlated with PTG. Acceptance–resignation was positively related to depression and anxiety and negatively related to PTG. Depression was negatively related to PTG. In addition, research has shown that both confrontation and avoidance are positively associated with acceptance–resignation. Anxiety was positively associated with depression.

The adjusted model indicated that social support affected PTG both directly (*β* = 0.564, *P* = 0.002) and indirectly (through coping strategies and emotional mediation). Specifically, a one-standard-deviation increase in social support was associated with a 0.564-standard-deviation increase in PTG. Indirect association: Social support is associated with increased avoidance (*β* = 0.199, *P* = 0.034) and acceptance–resignation (*β* = -0.315, *P* = 0.002), decreased depression (*β* = -0.123, *P* = 0.009) and increased PTG.

### 3.5 Mediating effect analysis

**Figure 3:**
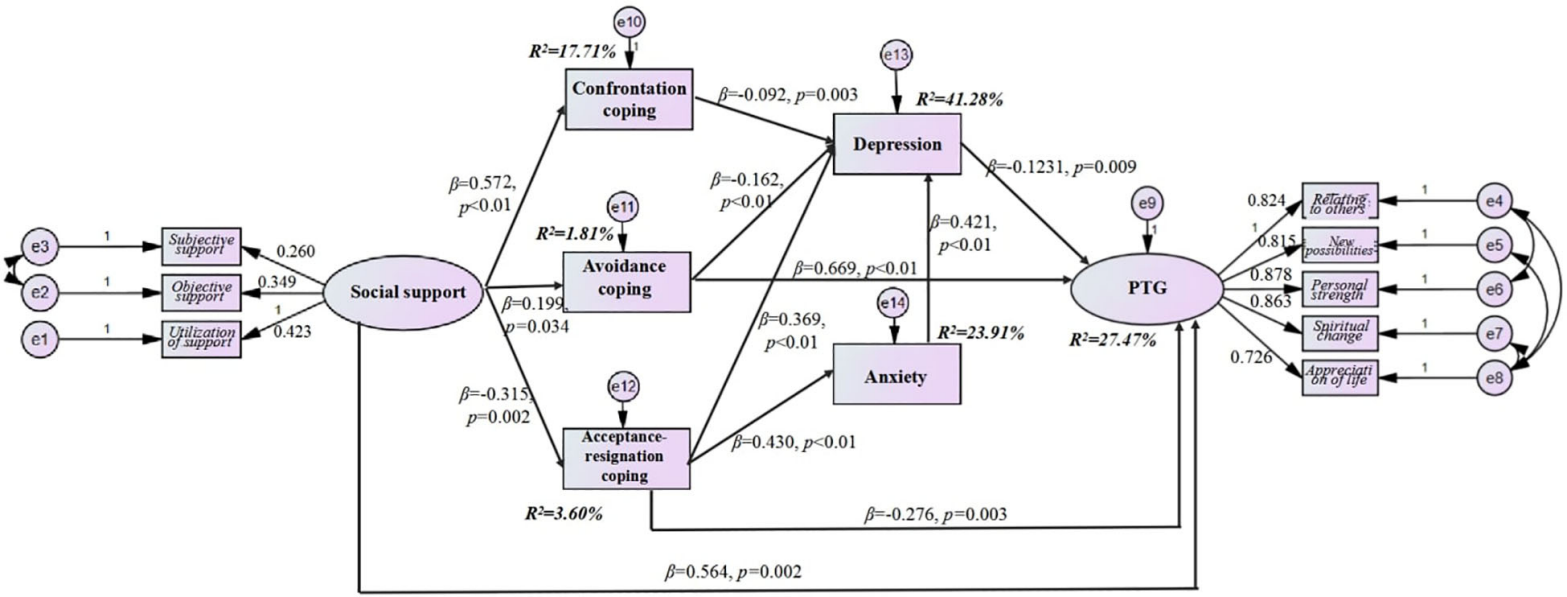
The adjustment model of social support, coping strategies, anxiety and depression, and PTG.

Path analysis revealed that social support was positively associated with PTG (*β* = 0.564, *P* = 0.004). The model diagram is shown in **Figure 3**. The solid arrows indicate significant paths (*P* < 0.05). The key paths include the following:

Social support → PTG: A direct positive path indicating that social support is a core predictor of PTG (*β* = 0.564).
Social support → Coping strategies: Social support promotes adaptive avoidance (*β* = 0.199) and suppresses acceptance–resignation (*β* = -0.315).
Coping strategies → Depression/Anxiety: Avoidance and confrontation strategies reduce depression (*β* = -0.092 and -0.162, respectively), whereas acceptance–resignation exacerbates depression (*β* = 0.369) and anxiety (*β* = 0.430).
Depression → PTG: Depression negatively impacts PTG through a negative path (*β* = -0.123), highlighting the importance of emotional management.

Social support indirectly affects PTG through the mediations of avoidance, acceptance–resignation, and the combination of acceptance–resignation, which affects depression through anxiety.

## 4 Discussion

### 4.1 H1: the relationship between social support and PTG

Our study revealed a positive association between social support and PTG in patients with hematological cancer (*β* =0.564, *P* = 0.002), which is consistent with the findings of Nenova et al. in patients undergoing hematopoietic stem cell transplantation (16). High social support may facilitate patients’ reappraisal of the meaning of illness by providing emotional companionship, informational resources, and tangible assistance (such as financial aid) (43, 44). Notably, while the direct association between social support and PTG has been widely reported (9), this study is the first to quantify the strength of this effect in patients with hematological cancer, suggesting that this patient group may be more sensitive to supportive resources than those with solid tumors (45). This finding underscores the necessity of systematically enhancing social support within clinical settings.

### 4.2 H2: the mediating role of coping strategies

Social support indirectly affects PTG through various coping strategies, but the directions of their effects are significantly different:

#### 4.2.1 Positive effect of avoidance coping

Avoidance coping has an independent positive effect on PTG (*β* = 0.669, *P* < 0.01), which is consistent with the conclusion drawn by Zhou et al. (10) in patients with gynecological cancer. A possible explanation is that the long-term nature of hematological cancer treatment (e.g., repeated chemotherapy) requires patients to engage in phased avoidance to relieve psychological stress, thereby providing a buffer for subsequent adaptive adjustment (23).

#### 4.2.2 Negative effect of acceptance–resignation coping

Acceptance–resignation coping significantly suppresses PTG (*β* = -0.276, *P* < 0.01), reflecting patients’ feelings of helplessness and hopelessness toward the disease (46). This result contrasts with the findings of Gao et al. (25) in colorectal cancer patients, which may be due to the high recurrence risk and invasive nature of hematological cancer treatment, exacerbating patients’ tendency toward passive coping. Additionally, acceptance–resignation may reflect the fatalistic attitudes toward illness rooted in traditional Chinese Taoist beliefs (47).

#### 4.2.3 Interactive effects of coping strategies

Social support promotes both avoidance and confrontation strategies (*β* = 0.199 and 0.572, respectively), indicating that these two strategies are not mutually exclusive but complementary adaptive mechanisms. For example, patients may obtain disease-related information through confrontation while temporarily detaching from stressful situations through avoidance to maintain psychological resilience (48).

### 4.3 H3-H4: the chain mediating effect of depression and anxiety

Depression plays a central mediating role between coping strategies and PTG (*β* = -0.123, *P* = 0.009), whereas the effect of anxiety is not significant (*β* = 0.116, *P* = 0.272). This finding challenges traditional perspectives (e.g., (28), in head and neck cancer patients), suggesting that in patients with hematological cancer, depression may be a more critical emotional target for intervention. Possible mechanisms include the deep psychological impact of depression. Depression is closely associated with reduced self-efficacy and cognitive rigidity (49), which may impede patients’ positive reconstruction of traumatic events. The dual nature of anxiety: Although anxiety is highly correlated with acceptance–resignation coping (*β* = 0.430), its direct effect on PTG is not significant. This may indicate that a certain level of anxiety can motivate patients to seek support (e.g., joining support groups), thereby offsetting its negative effects (29). Notably, the path assumptions in the mediation model (e.g., “social support → depression → PTG”) are based on the theoretical temporality of the variables. However, cross-sectional data cannot verify this sequence. Depression may be both a consequence of inadequate social support and a cause that leads to reduced social support (44).

### 4.4 H5: the relationship between social support and PTG operates through sequential mediation pathways involving coping strategies and subsequent affective states

Our findings reveal a critical sequential mediation pathway—”social support → coping strategies (avoidance/acceptance–resignation) → depression → PTG”. This multistage mechanism operates differently across coping dimensions:

#### 4.4.1 Avoidance–depression pathway

Social support enhances avoidance coping (*β* = 0.199), which paradoxically reduces depressive symptoms (*β* = -0.162). This aligns with Kunz et al.’s (23) observation that strategic disengagement may alleviate emotional exhaustion during prolonged treatments such as chemotherapy. However, this pathway’s adaptive value appears context dependent, requiring precise temporal modulation to prevent chronic avoidance.

#### 4.4.2 Acceptance–resignation exacerbation pathway

Reduced social support is correlated with increased acceptance–resignation (*β* = -0.315), which amplifies depression (*β* = 0.369) and subsequently diminishes PTG. This cascade reflects the cultural embeddedness of passive coping in Chinese healthcare contexts, where fatalistic beliefs (47) may interact with treatment-related uncertainties to reinforce resignation.

In addition, this study revealed a chain mediation pathway of “social support → avoidance/acceptance–resignation strategies → depression → PTG”, providing a new direction for psychological interventions across different cancer types. Our study extends existing theories in the following ways:

#### 4.4.3 Complexity of coping strategies

Unlike the traditional “dichotomy of coping strategies”, this study demonstrates that avoidance coping may have adaptive value in specific contexts (e.g., long-term treatment), calling for the inclusion of situational moderators in theoretical models.

#### 4.4.4 Mechanistic differences across cancer types

Compared with patients with solid tumors (e.g., 25), patients with hematological cancers, owing to the invasiveness of treatment and prognostic uncertainty, rely more strongly on the buffering role of social support and depression management for PTG formation. This provides a new perspective for research on heterogeneity in psychological responses to cancer.

#### 4.4.5 Cultural specificity

This study reveals that resignation coping is associated with the “fatalistic view” of Chinese culture (47), suggesting that future research should pay attention to the impact of cultural background on coping strategy selection.

## 5 Limitations

There are several limitations in this study. First, we examined only patients with hematologic cancers in a tertiary hospital in Sichuan Province, and there may be some regional differences and selection biases. For example, patients who participated in the survey may have had higher treatment adherence or stronger psychological resilience (e.g., patients who voluntarily participated in the study were more inclined to seek social support), whereas those who did not participate may have refused to complete the questionnaire owing to severe illness or emotional issues. The sample diversity needs to be expanded to verify the universality of the conclusions. Second, the cross-sectional nature of our study precludes the establishment of causal relationships. While we observed significant associations between social support and mental health outcomes, we cannot determine whether social support directly improves mental health or whether better mental health facilitates the perception and utilization of social support. The study did not assess patients’ anxiety or depression levels prior to cancer diagnosis and thus could not rule out the potential confounding effects of baseline psychological status on the outcomes. Future studies should employ longitudinal designs to track changes over time and establish temporal precedence. Third, the reliance on self-reported measures introduces potential biases, such as recall bias and social desirability bias, which may affect the accuracy of the data. Participants may not accurately remember past events or may report responses that they believe are socially acceptable, rather than their true experiences. In the future, the objectivity of the data can be improved by combining physiological indicators (such as cortisol levels) and behavioral observation methods.

## 6 Conclusions

This study revealed that social support can positively predict PTG in patients with hematologic cancers and that coping strategies, anxiety, and depression are mediating variables between social support and PTG in patients. The effects of different coping strategies vary avoidance and acceptance–resignation coping strategies, anxiety, and depression have cascading mediating effects.

## 7 Clinical implications

On the basis of the above findings, the following measures are recommended for clinical nursing:

### 7.1 Dynamic assessment of coping strategies

The MCMQ scale (37) should be used to assess patients’ coping tendencies at various stages of treatment, with a focus on resignation strategies (e.g., “I feel powerless”) and avoidance strategies (e.g., “I avoid talking about the disease”). For patients with a high tendency toward resignation, family-involved care, such as having family members record and report patients’ daily emotional changes to the nursing team, should be introduced.

### 7.2 Stratified psychological interventions

For avoidant patients, a “buffer period” support program, such as offering art therapy or short-term psychological counseling, should be implemented, followed by a gradual transition to disease adaptation training. Patients at high risk of depression should collaborate with psychiatrists to develop a combined pharmacological and psychological intervention plan, prioritizing mindfulness-based stress reduction (MBSR) to improve emotional resilience (50).

### 7.3 Optimization of social support networks

Establish a hospital-community linkage platform where recovered patients can serve as volunteers and share coping experiences through “peer support groups” (e.g., emotional management skills during chemotherapy). Mobile health technologies (e.g., WeChat mini-programs) should be used to deliver personalized support resources, such as automatically sending motivational messages or recovery stories the basis of patients’ treatment stages (51).

## Data Availability

The raw data supporting the conclusions of this article will be made available by the authors, without undue reservation.
